# Suppression of stacking-fault expansion in 4H-SiC PiN diodes using proton implantation to solve bipolar degradation

**DOI:** 10.1038/s41598-022-23691-y

**Published:** 2022-11-05

**Authors:** Masashi Kato, Ohga Watanabe, Toshiki Mii, Hitoshi Sakane, Shunta Harada

**Affiliations:** 1 Nagoya Institute of Technology, Gokiso, Showa, Nagoya, 466-8555 Japan; 2 SHI-ATEX Co. Ltd, 1501, Imazaike, Saijo, Ehime 799-1393 Japan; 3 Nagoya University, Furo, Chikusa, Nagoya, 464-8601 Japan

**Keywords:** Electrical and electronic engineering, Electronic devices

## Abstract

4H-SiC has been commercialized as a material for power semiconductor devices. However, the long-term reliability of 4H-SiC devices is a barrier to their widespread application, and the most important reliability issue in 4H-SiC devices is bipolar degradation. This degradation is caused by the expansion of single Shockley stacking-faults (1SSFs) from basal plane dislocations in the 4H-SiC crystal. Here, we present a method for suppressing the 1SSF expansion by proton implantation on a 4H-SiC epitaxial wafer. PiN diodes fabricated on a proton-implanted wafer show current–voltage characteristics similar to those of PiN diodes without proton implantation. In contrast, the expansion of 1SSFs is effectively suppressed in PiN diodes with proton implantation. Therefore, proton implantation into 4H-SiC epitaxial wafers is an effective method for suppressing bipolar degradation in 4H-SiC power-semiconductor devices while maintaining device performance. This result contributes to the development of highly reliable 4H-SiC devices.

## Introduction

Silicon carbide (SiC) is widely known as a semiconductor material for high power, high frequency semiconductor devices which can operate in harsh environment^1^. There are several polytypes in SiC, and, among the polytypes, 4H-SiC has superior physical properties for semiconductor devices, such as the high electron mobility and the high breakdown electric field^2^. 4H-SiC wafers with a 6-inch diameter are now commercialized and employed for the mass production of power semiconductor devices^3^. Traction systems in electric vehicles and trains have been fabricated using 4H-SiC power semiconductor devices^4,5^. However, 4H-SiC devices still have long-term reliability issues, such as dielectric breakdown or ruggedness in short-circuit connection^6,7^, and one of the most important reliability issues is bipolar degradation^2,8–11^. This bipolar degradation was discovered more than 20 years ago, and it has been a long-lasting issue for SiC device fabrication.

Bipolar degradation is caused by the expansion of single Shockley stacking-faults (1SSFs) from basal plane dislocations (BPDs) in 4H-SiC crystals by a recombination enhanced dislocation glide (REDG)^12–19^. Therefore, 4H-SiC power devices can be fabricated without bipolar degradation if the expansion of the BPDs is suppressed to 1SSF. Several suppression methods have been reported for the expansion of BPDs, such as the conversion of BPDs to threading edge dislocations (TEDs)^20–24^. In recent SiC epitaxial wafers, BPDs are mostly present in the substrates but not in the epilayers, owing to the conversion of BPDs to TEDs in the initial stage of epitaxial growth^20–24^. Therefore, a remaining issue for bipolar degradation is the expansion of BPDs in substrates^25–27^. Inserting a “recombination enhancing layer” between a drift layer and a substrate has been suggested as an effective method to suppress the expansion of BPDs in the substrate^28–31^. This layer enhances the recombination probability of electron–hole pairs in the epitaxial layer and decreases the number of electron–hole pairs at the BPDs in the SiC substrate. The reduction of electron–hole pairs decreases the driving force of REDG for BPDs in the substrate, and thus the recombination enhancing layer can suppress bipolar degradation. Notably, the layer insertion incurs an additional cost in wafer production, while, without the layer insertion, it is difficult to decrease the number of electron–hole pairs only by controlling the carrier lifetime control^32^. Thus, there are still strong requirements for the development of other suppression methods to achieve a better balance between the device fabrication costs and yield.

Because the expansion of BPDs to 1SSFs requires the movement of partial dislocations (PDs), the pinning of PDs is a promising method for the suppression of bipolar degradation. Although the pinning of PDs by metal impurities has been reported^33^, the BPDs in 4H-SiC substrates are more than 5 µm away from the epilayer surfaces. Moreover, because the diffusion coefficients of any metal in SiC are very small, it is difficult to diffuse the metal impurities into the substrates^34^. The ion implantation of metals is also difficult because of the relatively large atomic mass of metals^35^. In contrast, in the case of the lightest element, hydrogen, an ion (proton) can be implanted at a depth of more than 10 µm in 4H-SiC using a MeV-class accelerator. Therefore, if proton implantation affects the pinning of PDs, then it can be used to suppress the expansion of BPDs in the substrates^36^. However, proton implantation can damage 4H-SiC and result in the deterioration of device performance^37–40^.

To overcome the deterioration of device performance by proton implantation, high-temperature annealing, which is similar to the annealing method commonly used after acceptor-ion implantation in device processing, is used to recover the damage^1,40–42^. Although it has been reported that the outdiffusion of hydrogen by high-temperature annealing is observed through secondary ion mass spectrometry (SIMS)^43^, there is a possibility that only hydrogen atoms near the PDs, which are not dense enough for detection by SIMS, affect the pinning of PDs. Therefore, in this study, we implanted a proton onto a 4H-SiC epitaxial wafer before the device-fabrication process, which includes high-temperature annealing. We adopted PiN diodes as trial-device structures and fabricated them on a proton-implanted 4H-SiC epitaxial wafer. We then observed the current–voltage characteristics to examine the deterioration of the device performance due to proton implantation. Subsequently, we observed 1SSFs expansion in electroluminescence (EL) images after applying electrical stress to the PiN diodes. Finally, we confirmed the effects of proton implantation on the suppression of 1SSF expansion.

## Results

**Figure 1:**
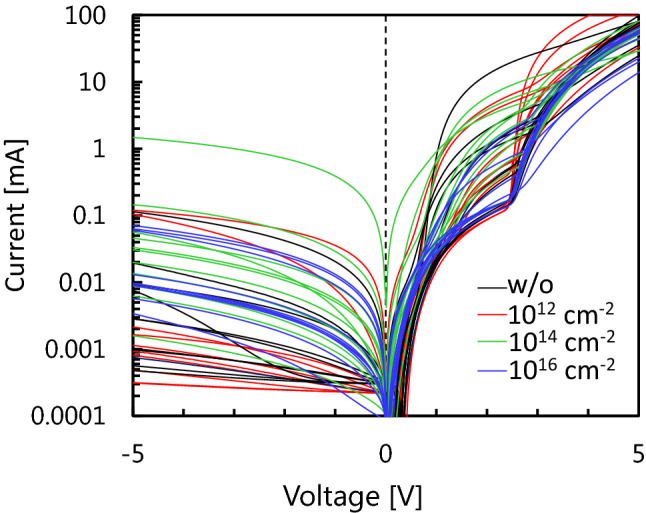
Current–voltage characteristics for the PiN diodes with and without proton implantation at room temperature. The legend indicates the proton doses.

**Figure 2:**
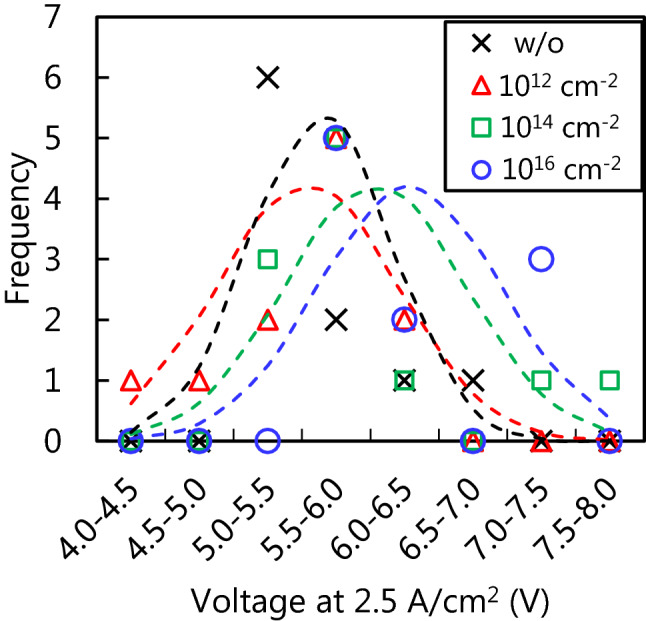
Frequency of the voltages at a forward current of 2.5 A/cm^2^ for the PiN diodes with and without proton implantation. The dotted lines are fitting with the normal distribution.

The current–voltage (I-V) characteristics of the PiN diodes at room temperature in regions with and without proton implantation before the pulsed-current stress are shown in Fig. 1. PiN diodes with proton implantation show rectifying properties similar to those without proton implantation, even though the I-V characteristics among the diodes are scattered. To delineate the difference among the implantation conditions, we plotted frequencies of voltages at a forward current density of 2.5 A/cm^2^ (corresponding to 100 mA) to statistically as shown in Fig. 2. The curves fitted by the normal distribution are also indicated by the dotted lines. As illustrated by the peaks of the curves, the on-state resistance slightly increased with proton doses of 10^14^ and 10^16^ cm^−2^, whereas the PiN diodes with proton doses of 10^12^ cm^−2^ showed almost the same performance as those without proton implantation. We also performed proton implantation after PiN diode fabrication, and the diodes did not exhibit uniform EL, as shown in Fig. S1, due to the damage caused by the proton implantation, as reported in previous studies^37–39^. Therefore, annealing at 1600 °C after Al ion implantation which is an essential process for device fabrication to activate Al acceptor recovered the damages induced by proton implantation, resulting in similar I-V characteristics between the PiN diodes with and without proton implantation. The frequency of the reverse current at − 5 V is also plotted in Fig. S2, and no significant difference was observed between the diodes with and without proton implantation.

**Figure 3:**
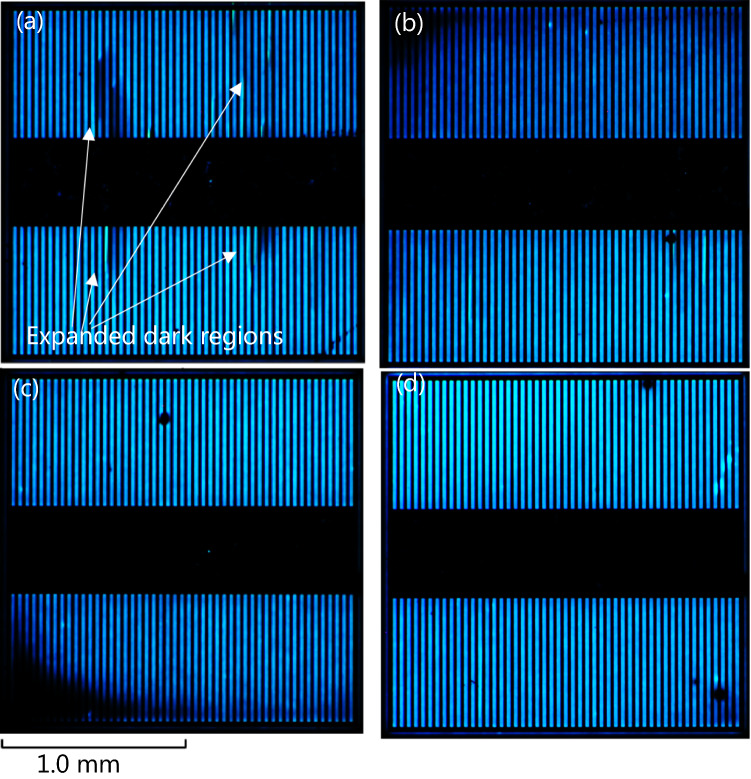
EL images of PiN diodes at 25 A/cm^2^ after the electrical stress with 2 h for (**a**) without proton implantation and when implanted with (**b**) 10^12^ cm^−2^, (**c**) 10^14^ cm^−2^, and (**d**) 10^16^ cm^−2^ doses of protons.

**Figure 4:**
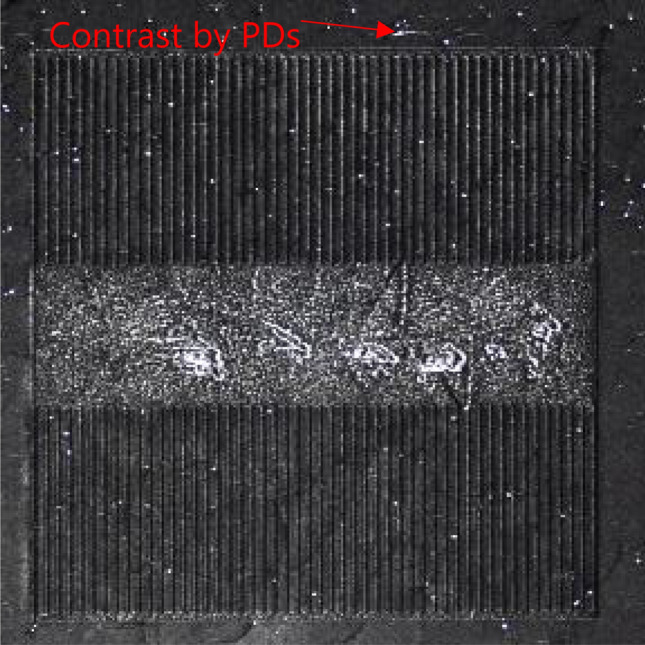
X-ray topographic image of the PiN diode shown in Fig. 3a.

EL images of the PiN diodes at a current density of 25 A/cm^2^ after electrical stress are shown in Fig. 3. Before the pulsed-current stress is applied, no dark region is observed for any of the diodes, as shown in Fig. S2. However, as shown in Fig. 3a, after applying electrical stress, several bar-shaped dark regions with bright edges are observed in the PiN diode without proton implantation. Such bar-shaped dark regions in EL images were observed for 1SSFs expanded from BPDs in the substrates^28,29^. In contrast, a few extended stacking faults were observed in the proton-implanted PiN diodes, as shown in Fig. 3b–d. Using X-ray topography, we confirmed the presence of PDs that could be moved from the BPDs in the substrate at the periphery of the contact in the PiN diode without proton implantation (Fig. 4: this image was taken without removal of the top electrode, and PDs under the electrode are invisible). Therefore, the dark regions in the EL images correspond to the expanded 1SSFs from the BPDs in the substrate. The EL images of the other stressed PiN diodes are shown in Figs. S3–S6 and videos with and without expansion of the dark region (Time changes in EL images for the PiN diodes without proton implantation and with implantation of 10^14^ cm^−2^) are also shown in the supplementary information.

**Figure 5:**
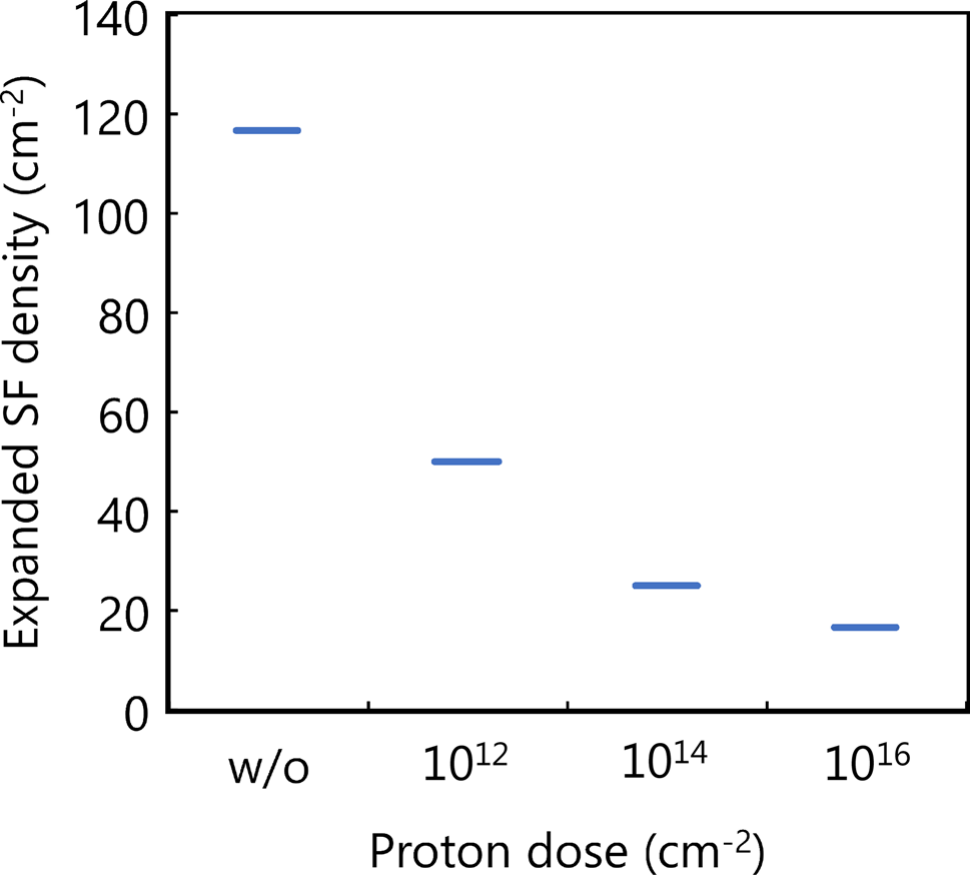
Expanded SF densities for the PiN diodes with and without proton implantation after the pulsed-current stress (each condition includes three stressed diodes).

We calculated the density of the expanded 1SSFs by counting the dark regions with a bright edge in the three PiN diodes for each condition, as shown in Fig. 5. The expanded 1SSF densities decreased with increasing proton doses, and even at a dose of 10^12^ cm^−2^, the density of the expanded 1SSF was significantly lower than that of PiN diodes without proton implantation.

The reduction in carrier lifetime also influences the suppression of expansion, and proton implantation reduces carrier lifetime^32,36^. We observed carrier lifetimes in a 60 µm-thick epitaxial layer with 10^14^ cm^−2^ proton implantation. From the initial carrier lifetime, although implantation reduced the value to ~ 10%, subsequent annealing recovered it to ~ 50%, as shown in Fig. S7. Therefore, the reduced carrier lifetime owing to proton implantation was recovered by high temperature annealing. Although 50% reduction of the carrier lifetime may also have suppression of the stacking fault expansion, I-V characteristics, which generally depend on the carrier lifetime, among the diodes with and without implantation show only slight differences. Therefore, we consider that pinning of PDs play a role for suppression of 1SSF expansion.

Although no hydrogen was detected by SIMS after annealing at 1600 °C, as reported in a previous study^43^, we observed the effects of proton implantation on the suppression of 1SSF expansion, as shown in Figs. 3, 4. Therefore, we consider that PDs were pinned by hydrogen atoms that had a density lower than the detection limit of SIMS (2 × 10^16^ cm^−3^) or point defects introduced by implantation. It should be noted that we did not confirm an increase in the on-resistance due to the expanded 1SSF after the pulsed-current stress. This is possibly due to imperfect ohmic contacts fabricated using our process, which will be solved in the near future.

In summary, we developed a suppression method for the expansion of BPDs to 1SSFs in 4H-SiC PiN diodes using proton implantation before device fabrication. The deterioration of the I-V characteristics by proton implantation was not significant, particularly at a proton dose of 10^12^ cm^−2^; however, the effect of the suppression of 1SSF expansion was significant. Although we fabricated 10 µm thick PiN diodes with 10 µm deep proton implantation in this study, there is a possibility of the further optimization for implantation conditions and application to the fabrication of other types 4H-SiC devices. Additional device-fabrication costs during proton implantation should be considered, but they will be similar to the Al-ion implantation costs, which is an essential process for the fabrication of 4H-SiC power devices. Therefore, proton implantation prior to device processing is a potential method for fabricating bipolar degradation-free 4H-SiC power devices.

## Methods

### Sample preparation

A 4-inch n-type 4H-SiC wafer with an epitaxial layer thickness of 10 µm and a donor doping concentration of 1 × 10^16^ cm^−3^ was used as the sample. H^+^ ions were implanted into the wafer to a depth of ~ 10 µm using an acceleration energy of 0.95 MeV at room temperature with a normal angle to the wafer surface before device processing. The wafer had sections without and with proton doses of 10^12^, 10^14^ or 10^16^ cm^−2^ using a mask on the wafer at proton implantation. Then, an Al ion with proton doses of 10^20^ and 10^17^ cm^−3^ was implanted on the entire wafer at depths of 0–0.2 µm and 0.2–0.5 µm from the surface, respectively, and subsequent annealing was performed at 1600 °C with a carbon cap to form a p-type layer. Subsequently, the backside Ni contacts were deposited on the substrate side, while 2.0 mm × 2.0 mm comb-shaped Ti/Al front contacts which shape was formed by photolithography and a lift-off process were deposited on the epilayer side. Finally, contact annealing was conducted at 700 °C. After dicing the wafer to chips, we performed characterization and stress applications.

### Characterization and stress application

I-V characteristics of the fabricated PiN diodes were observed using an HP4155B semiconductor-parameter analyzer. As an electrical stress, 10-ms-long pulsed-currents of 212.5 A/cm^2^ were injected at a frequency of 10 pulses/s for 2 h. When we employed lower current density or frequency, we have not observed 1SSF expansion even in the PiN diodes without proton implantation. During electrical-stress application, the temperature of the PiN diode was ~ 70 °C without intentional heating as shown in Fig. S8. EL images were obtained at a current density of 25 A/cm^2^ before and after electrical stress. Grazing incidence synchrotron reflection X-ray topography was performed using a monochromatic X-ray beam (λ = 0.15 nm) with a **g** vector of − 1–128 or 11–28 at BL8S2, in the Aichi Synchrotron Radiation Center (details can be found in Ref.^44^).

### Statistics

From the I-V characteristics for each condition of the PiN diodes, frequency of the voltages at a forward current density of 2.5 A/cm^2^ were extracted with a 0.5 V interval in Fig. 2. From averages of the voltages *V*_ave_ and standard deviations of the voltages *σ*, we drew the curves with normal distribution as the dotted lines in Fig. 2 using the following equation:

1 $$\mathrm{Frequency }\left(V\right)=\frac{1}{\sqrt{2\pi {\sigma }^{2}}}\mathrm{exp}\left(-\frac{(V-{V}_{ave}{)}^{2}}{2{\sigma }^{2}}\right).$$

## Supplementary Information

Supplementary Video 1.

Supplementary Video 2.

Supplementary Figures.

## Data Availability

All the relevant data are available from the corresponding authors upon reasonable request.
