# Supplementary material for: Suppression of stacking-fault expansion in 4H-SiC PiN diodes using proton implantation to solve bipolar degradation
Source: Sci Rep. 2022 Nov 5;12:18790. doi: 10.1038/s41598-022-23691-y (PMC9637098; doi:10.1038/s41598-022-23691-y)
Supplement: Supplementary file 3 — Supplementary Figures. [file 41598_2022_23691_MOESM3_ESM.docx]

Supplementary Information

**Suppression of stacking fault expansion in 4H-SiC PiN diodes by proton implantation to solve bipolar degradation**

Masashi Kato*, Ohga Watanabe, Toshiki Mii, Hitoshi Sakane, Shunta Harada


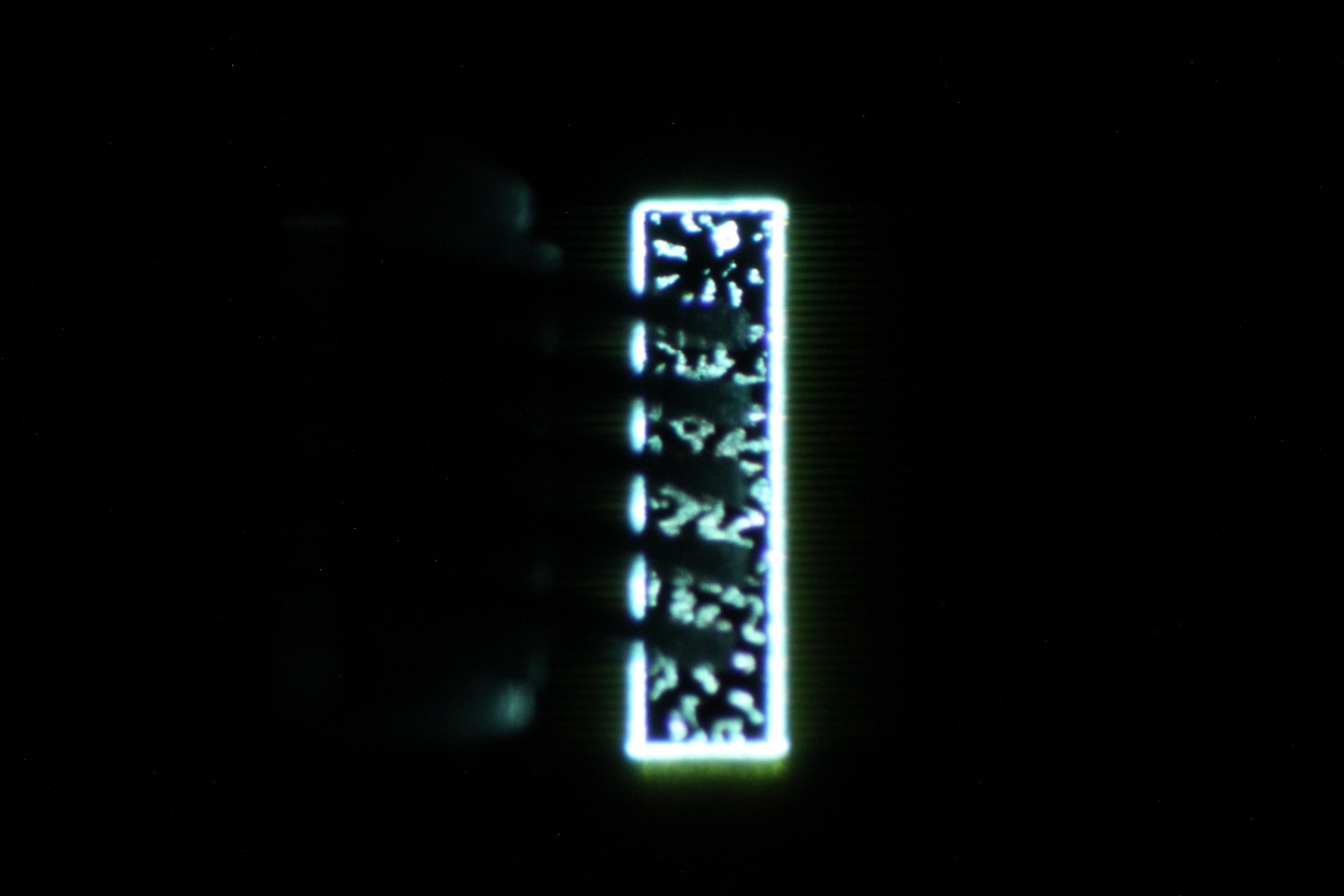


Fig. S1. EL image of a PiN diode with proton implantation after the fabrication processes at 125 A/cm^2^.

Fig. S2. Frequency of the reverse current at -5 V for the PiN diodes with and without proton implantation.


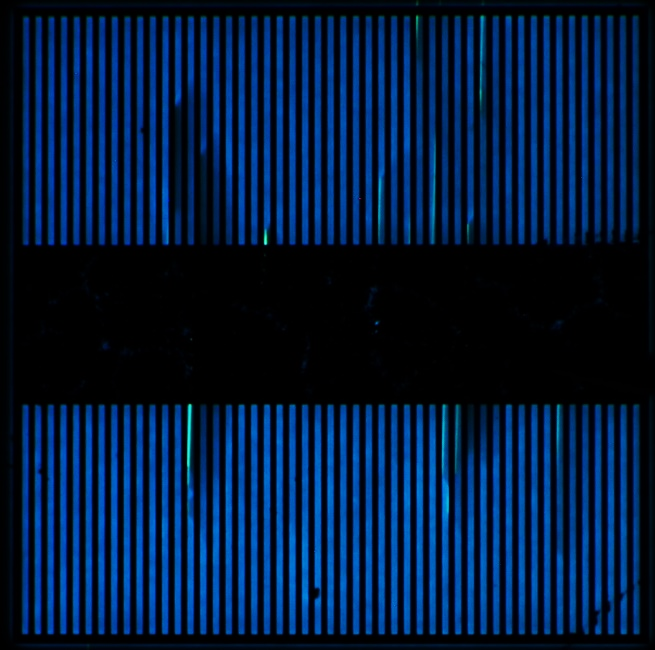

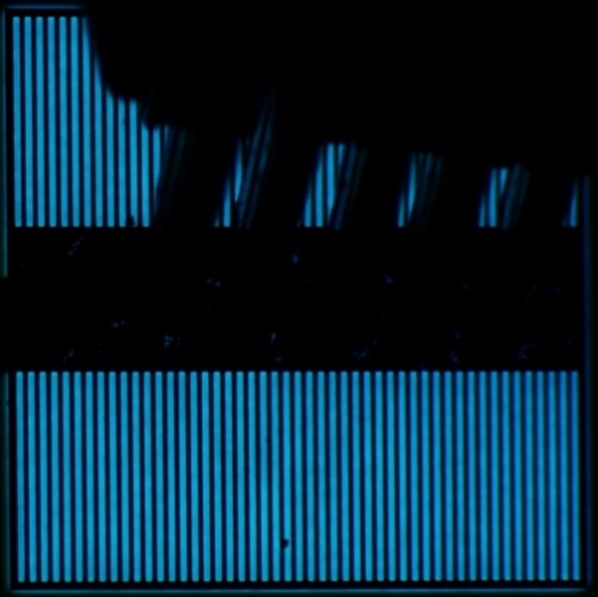


(b)

(a)


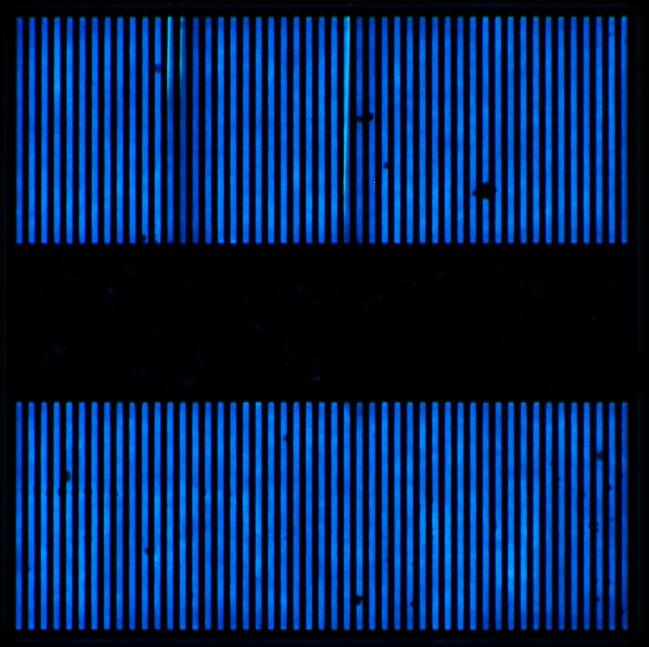

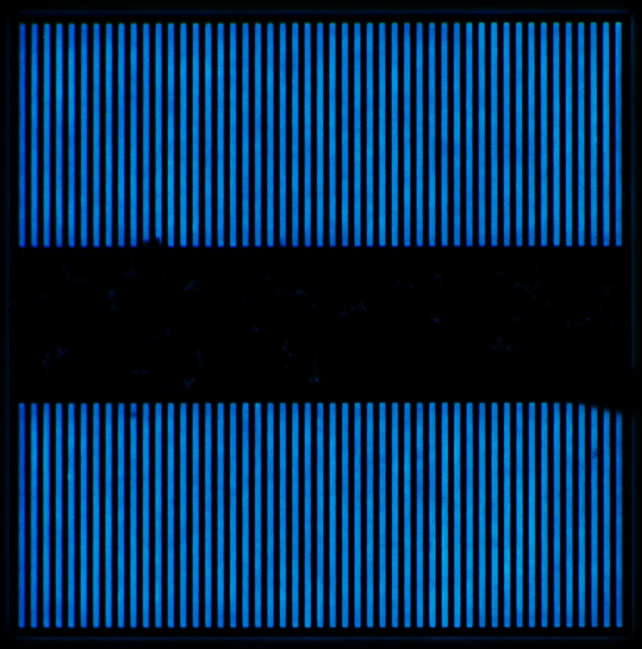


(c)

(d)

(e)

(f)


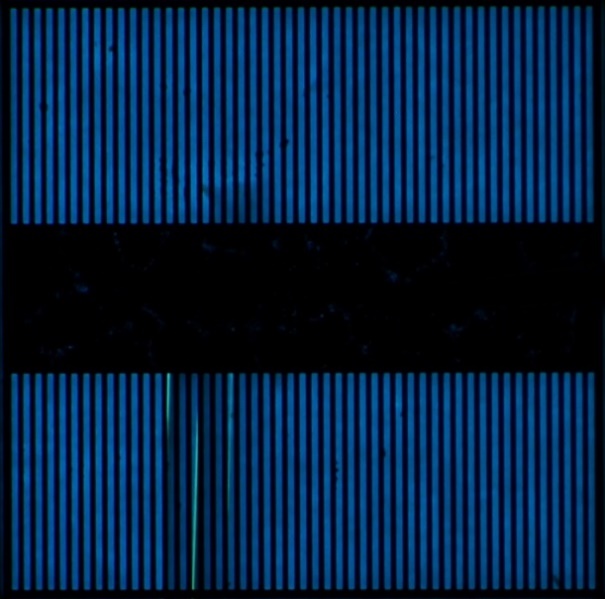

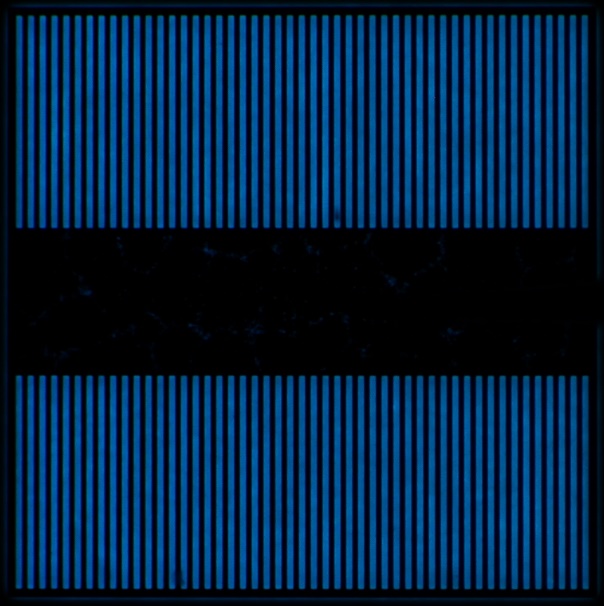


Fig. S3. EL images of PiN diodes w/o proton implantation at 25 A/cm^2^: before (a), (c) and (e), and after (b), (d) and (f) the electrical stress. (a) and (b) are for a first test chip, (c) and (d) are for a second test chip, (e) and (f) are for a third test chip, and (b) corresponds to Fig. 3(a). The wide dark region in (a) is due to presence of a probe, and, for other images, we employed a narrow probe to eliminate the dark region. The white arrows indicate location of the bright edge for the expanded 1SSFs.


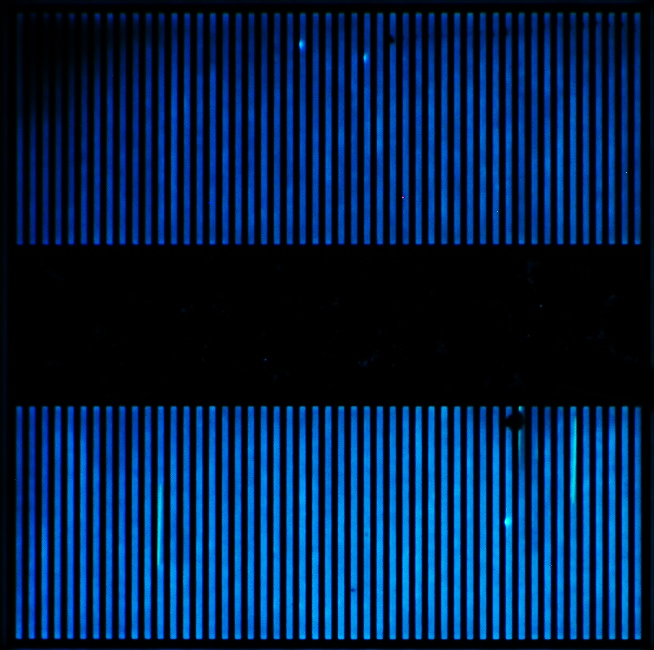

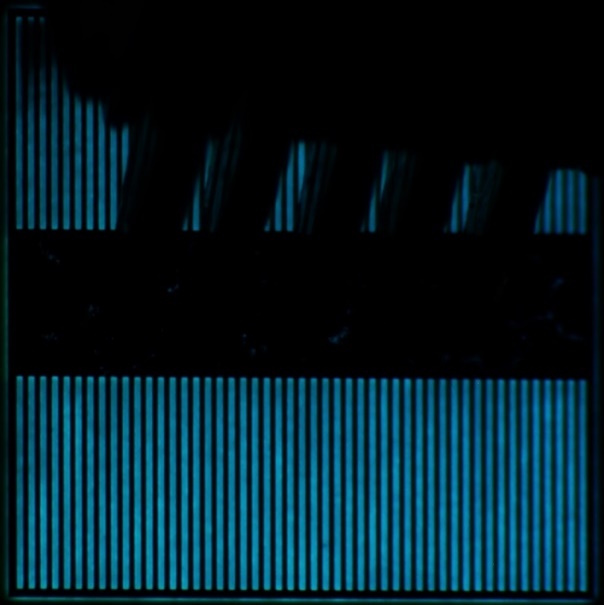


(b)

(a)


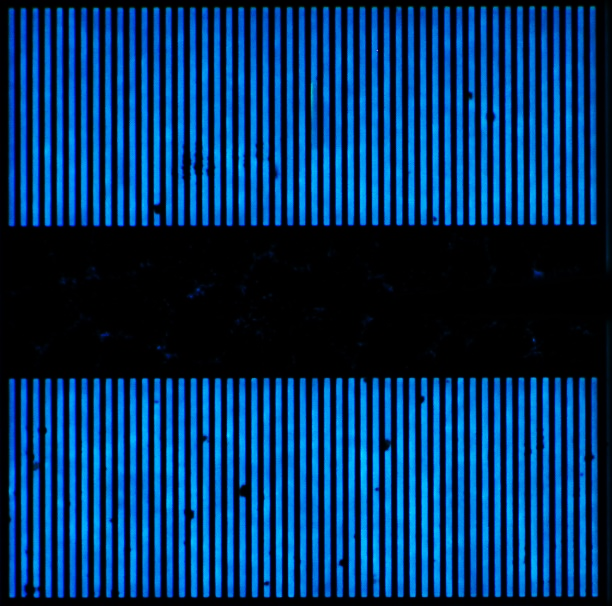

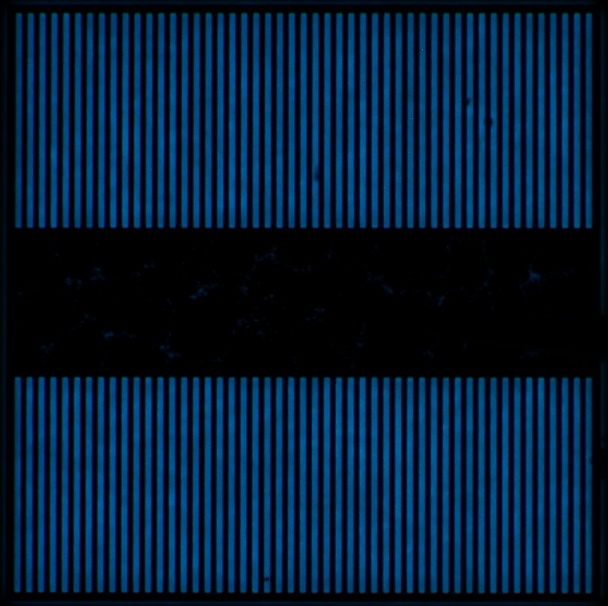

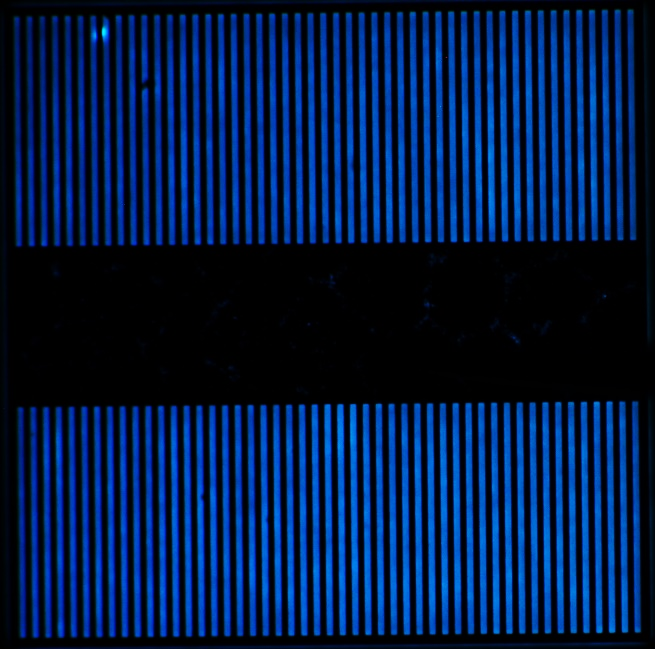

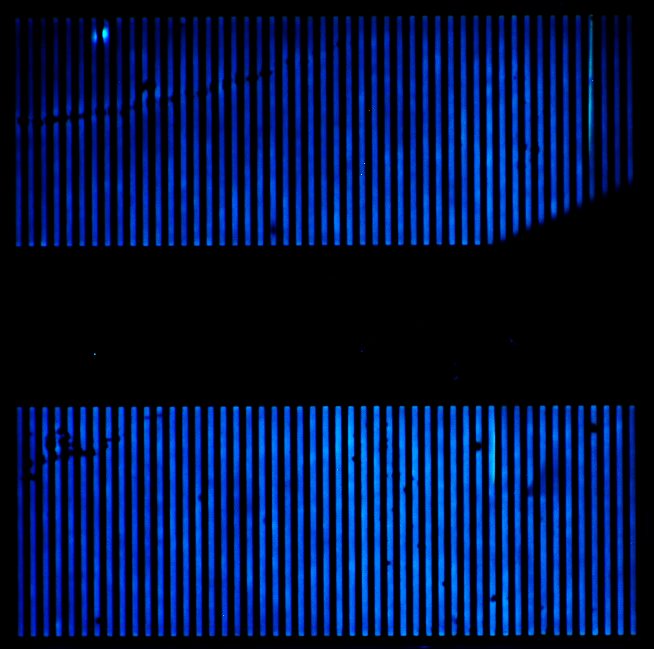


(c)

(d)

(f)

(e)

Fig. S4. EL images of PiN diodes with 10^12^ cm^-2^ proton implantation at 25 A/cm^2^: before (a), (c) and (e), and after (b), (d) and (f) the electrical stress. (a) and (b) are for a first test chip, (c) and (d) are for a second test chip, (e) and (f) are for a third test chip, and (b) corresponds to Fig. 3(b).


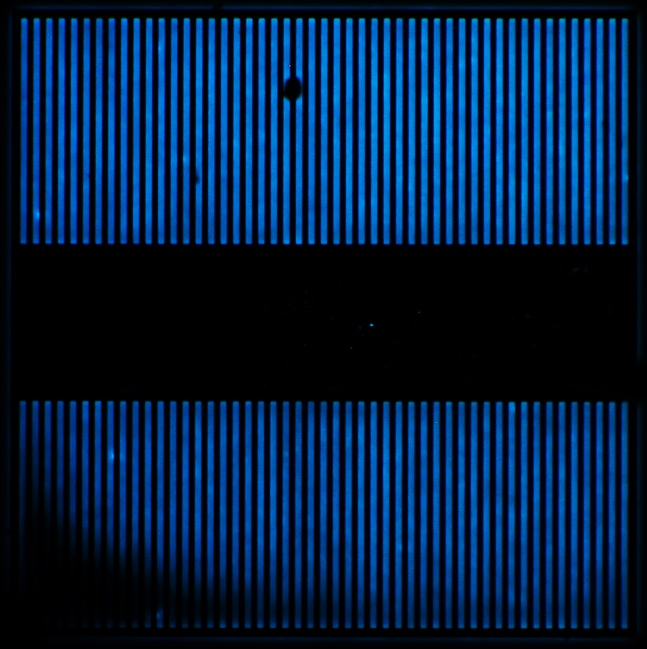

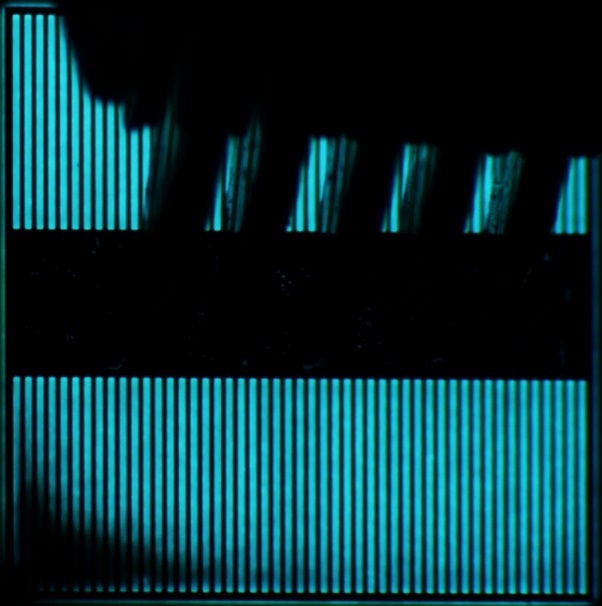


(b)

(a)


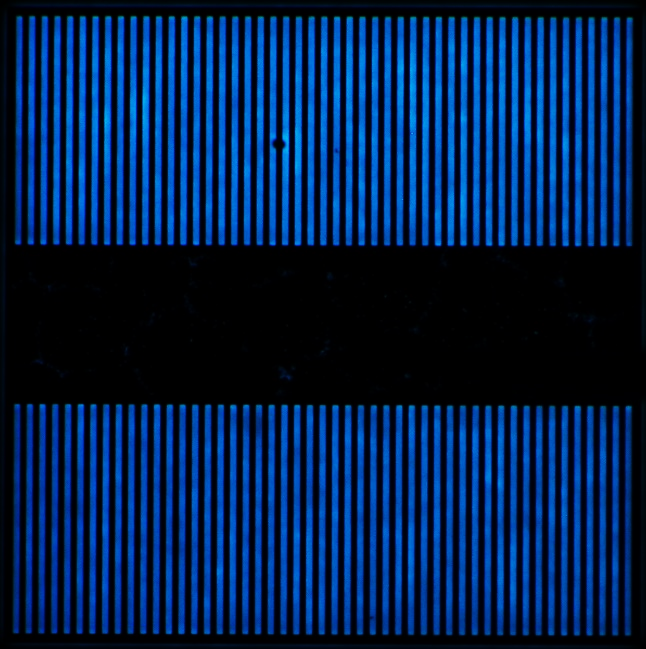

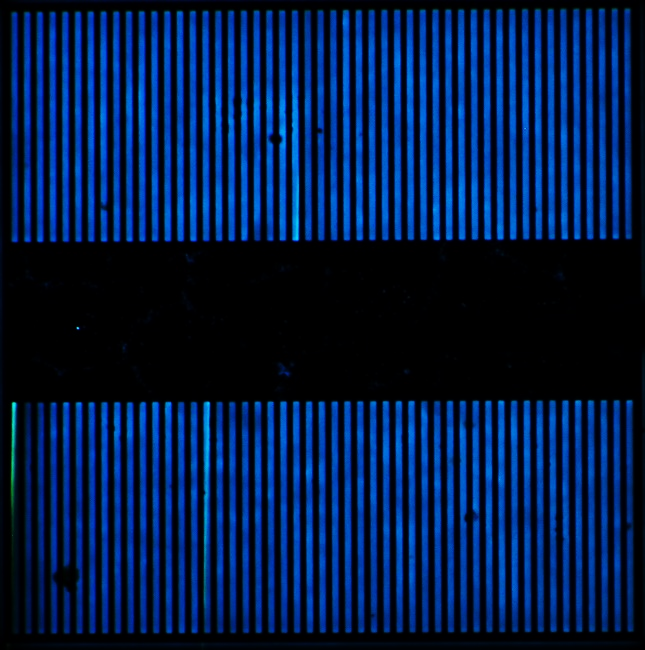


(d)

(c)


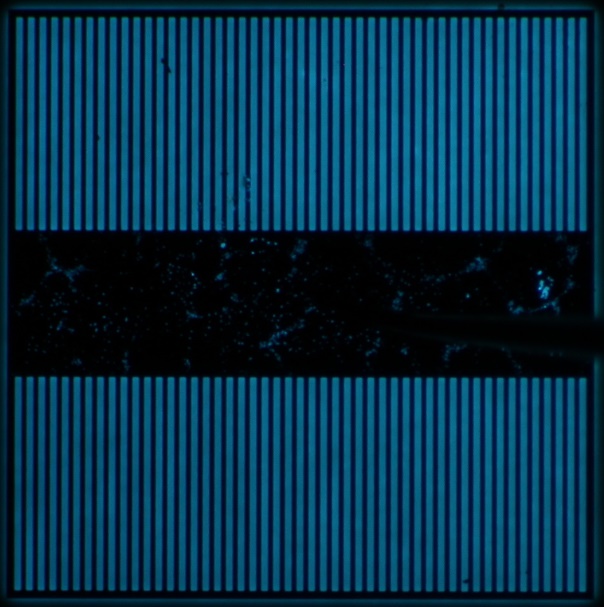

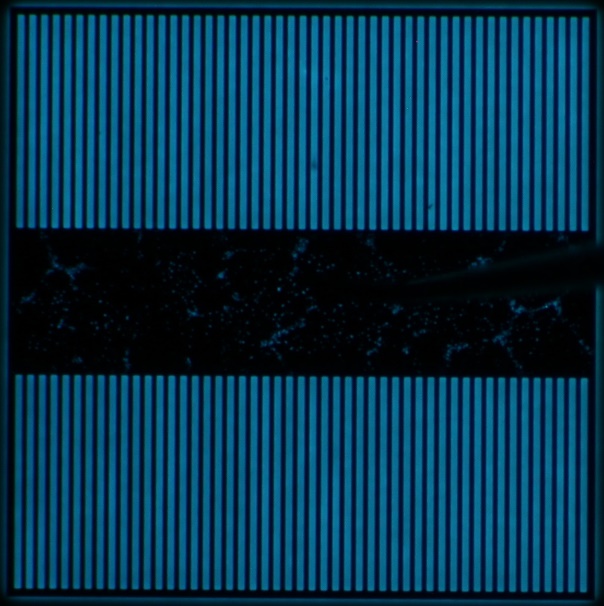


(f)

(e)

Fig. S5. EL images of PiN diodes with 10^14^ cm^-2^ proton implantation at 25 A/cm^2^: before (a), (c) and (e), and after (b), (d) and (f) the electrical stress. (a) and (b) are for a first test chip, (c) and (d) are for a second test chip, (e) and (f) are for a third test chip, and (b) corresponds to Fig. 3(c).


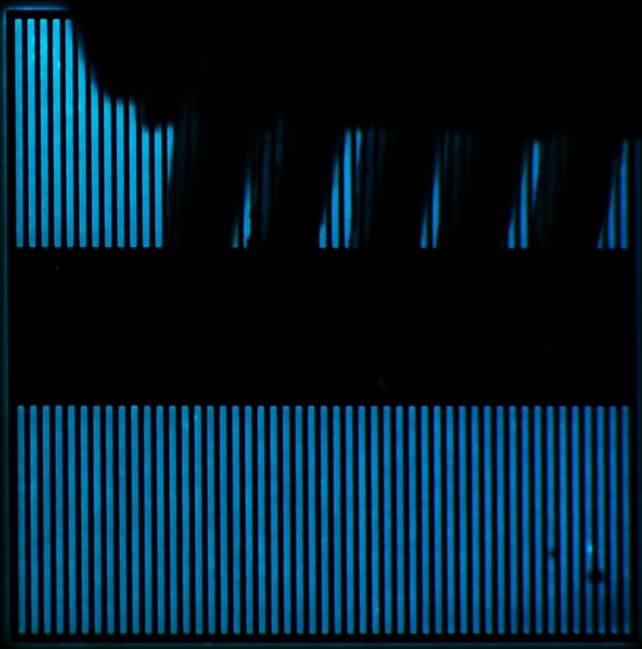

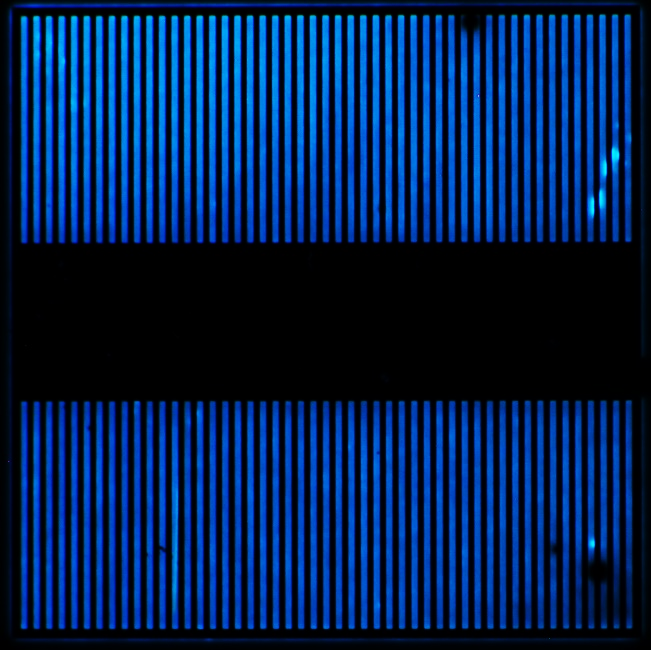


(b)

(a)


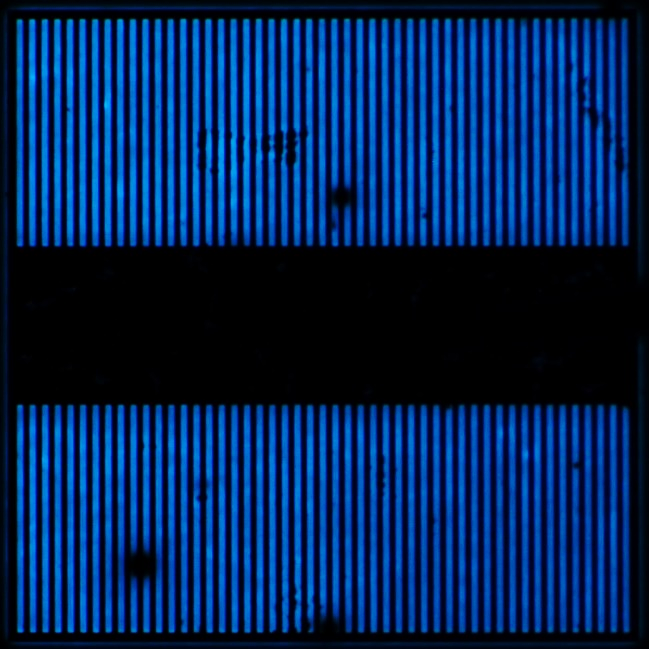

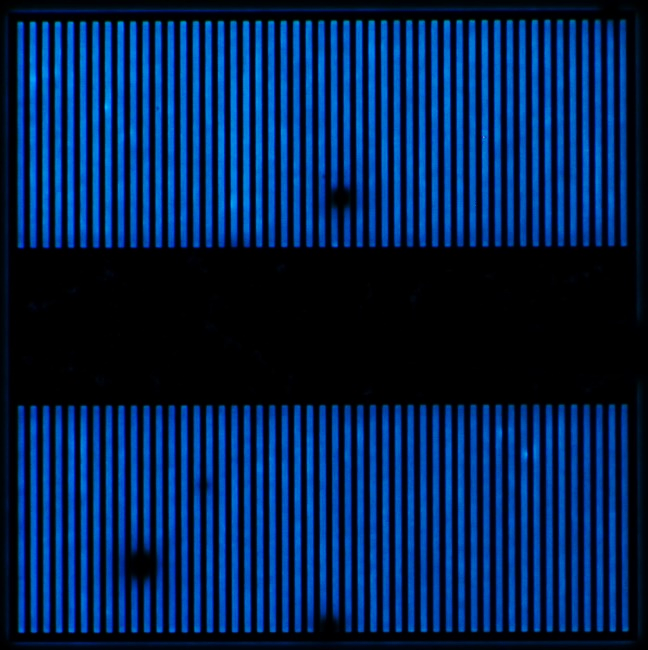


(c)

(d)


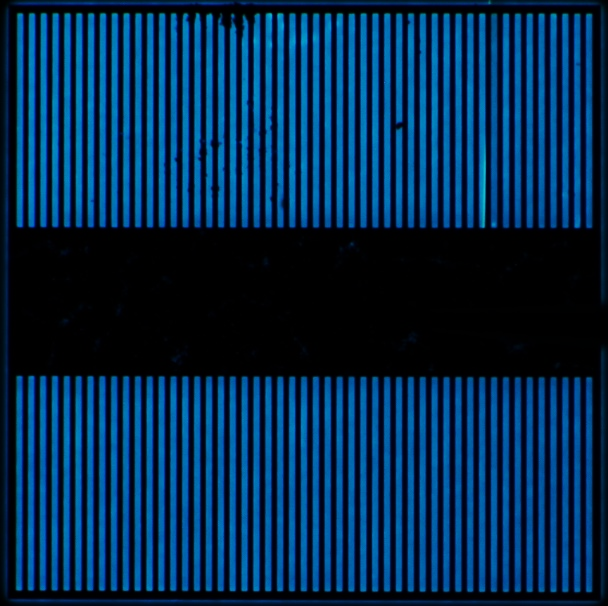

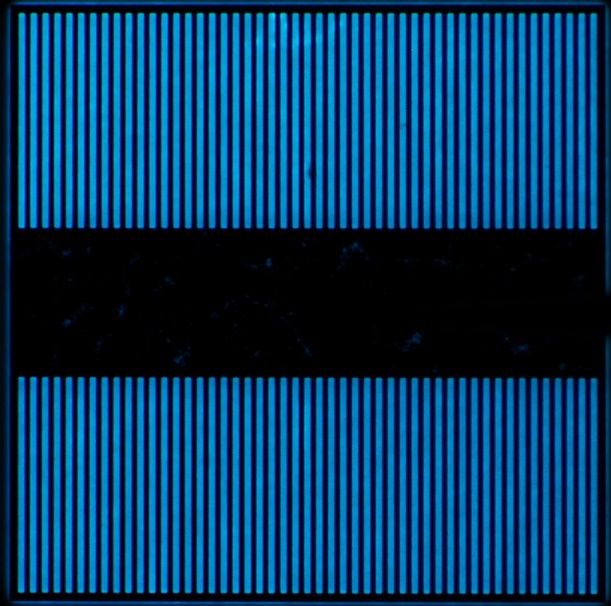


(e)

(f)

Fig. S6. EL images of PiN diodes with 10^16^ cm^-2^ proton implantation at 25 A/cm^2^: before (a), (c) and (e), and after (b), (d) and (f) the electrical stress. (a) and (b) are for a first test chip, (c) and (d) are for a second test chip, (e) and (f) are for a third test chip, and (b) corresponds to Fig. 3(d).

Fig. S7. Carrier lifetime (time from peak to 1/e of the microwave photoconductivity decay signal) observed from 60 μm epilayers (with a donor concentration of 10^15^ cm^-3^) with and without proton implantation and annealing. The proton implantation energy and dose were 1 MeV and 10^14^ cm^-2^. The excitation wavelengths of 266 nm corresponding to photon penetration depth of 1.2 μm was employed in the measurements.

Fig. S8. Typical PiN diode temperature during application of the electrical stress measured by thermocouple on the diode. This temperature was not controlled intentionally.
